# A Review of the Effects of Collagen Treatment in Clinical Studies

**DOI:** 10.3390/polym13223868

**Published:** 2021-11-09

**Authors:** Hsiuying Wang

**Affiliations:** Institute of Statistics, National Yang Ming Chiao Tung University, Hsinchu 30010, Taiwan; wang@stat.nycu.edu.tw

**Keywords:** clinical studies, collagen, scaffold, tissue engineering, treatment

## Abstract

Collagen, an abundant extracellular matrix protein, has been found to have a lot of pharmaceuticals, medicine, food, and cosmetics applications. Increased knowledge of collagen sources, extraction techniques, structure, and properties in the last decades has helped develop more collagen-based products and tissue engineering biomaterials. Collagen products have been playing an important role in benefiting the health of the human body, especially for aging people. In this paper, the effects of collagen treatment in different clinical studies including skin regeneration, bone defects, sarcopenia, wound healing, dental therapy, gastroesophageal reflux, osteoarthritis, and rheumatoid arthritis have been reviewed. The collagen treatments were significant in these clinical studies. In addition, the associations between these diseases were discussed. The comorbidity of these diseases might be closely related to collagen deficiency, and collagen treatment might be a good choice when a patient has more than one of these diseases, including the coronavirus disease 2019 (COVID-19). It concludes that collagen-based medication is useful in treating comorbid diseases and preventing complications.

## 1. Introduction

Collagen, an important protein produced by the body, is the main structural protein found in the skin, tendon, and bone. The word collagen originates from a Greek word that “kola” means gum and “gen” means producing. Collagen is considered to be one of the most useful biomaterials. Due to its low immunogenicity and high biocompatibility, it has been extensively studied as a polymer for use in many biomedical goods such as cosmetic and pharmaceutical products [1,2]. It also has been used as a safe and effective biomaterial in tissue engineering and clinical applications. It is an ingredient in dental composites, skin regeneration templates, and biodegradable matrices, and it has been used in cardiovascular surgery, plastic surgery, orthopedics, urology, neurology, and ophthalmology. There is a large demand for collagen in the food industry because it has high protein content and good functional properties such as water absorption capacity and the ability to form emulsions [3,4].

**Figure 1:**
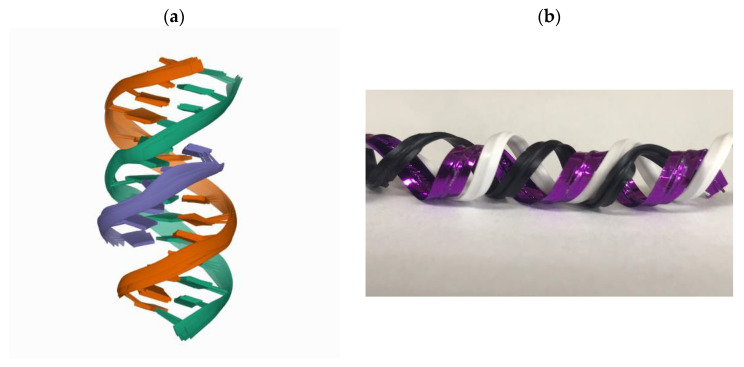
(**a**) A triplex helix structure plotted from RCSB protein data bank (https://www.rcsb.org/ accessed on 1 May 2021). (**b**) A triplex helix structure model.

Collagen is one of the most abundant proteins in many living organisms because it plays a connective role in biological structures. It is also the most abundant protein in the extracellular matrix (ECM). ECM is a non-cellular component within all tissues and organs and is a structural scaffold that can direct cell adhesion and migration, and regulate cellular growth and metabolism [5]. In addition to blood cells, other cells in human tissues are residing in ECM. Collagen fibrils form the main tension-resisting element of a complicated fiber-composite system in the ECM [6]. There are four structural levels of a collagen protein including primary structure (amino acid triplet), secondary structure (the α-helix), tertiary structure (triple helix), and quaternary structure (fibrils) [7]. It is formed in a triplex helix by three α chains (Figure 1). The chains are distorted around each other to form a tight and stable structure [8].

According to the α-chain composition, there are different types of collagens. About 28 types of collagen have been identified, but the dominant collagen is collagen type I. Over 90% of the collagen in the human body is collagen type I because of its wide prevalence in almost all connective tissues [9]. There are 5 most common types of collagen, type I–V [10,11]. Table 1 lists some functions of these five types of collagen. Collagen type I is the main component of the calcified tissue of teeth and bone and is present in skin, tendons, vasculature, lungs, and heart. It can be a ligand for receptor-mediated signalings such as integrins, OSCAR, GPVI, G6b-B, DDR1 and 2, and LAIR-1 of the leukocyte receptor complex [12]. Collagen type II is an abundant matrix molecule of cartilage and is associated with many diseases such as skeletal dysplasias, rheumatoid arthritis (RA) and osteoarthritis (OA) [13,14,15].

Collagen type III, consisting of only one collagen α chain, belongs to the fibrillar collagen group. It is a homotrimer composed of three identical α1(III) chains supercoiled around each other in a right-handed triple helix. It also is an important component of blood vessels and muscle. It appears to function along with type I collagen in the skin, ligament, tendon, periodontal ligament, vascular walls, and synovial membranes [16]. Collagen type III is related to vascular deficiency, aortic and arterial aneurysms, and Ehlers Danlos syndrome (EDS) [17]. EDS comprises ten types. EDS IV is the most severe type that is caused by mutations in a collagen type III corresponding gene COL3A1 [18]. Collagen type IV is the predominant collagen of the basement membrane forming the backbone of the basement membrane. Mutations in collagen type IV can cause chronic kidney disease, Alport’s syndrome [19]. Collagen type V is present in the corneal stroma, bone matrix, and the interstitial matrix of muscles, lungs, liver, and placenta [20]. Collagen type V functioned along with collagen type I in skin and tendon, and mutations in collagen type V had been shown to underlie classical EDS [21].

In this review paper, I aim to review the collagen-based treatment for several related issues including skin regeneration, bone defects, sarcopenia, wound healing, dental therapy, gastroesophageal reflux (GERD), OA and RA. In addition to these diseases, the relationship between GERD and coronavirus disease 2019 (COVID-19) is discussed. The collagen treatment for GERD in COVID-19 is suggested as an alternative to some other GERD drugs.

## 2. Source

At present, collagen can be extracted from natural animal and plant sources or be obtained from recombinant protein production systems using bacteria, yeast, insects or plants, mammalian cells, or artificial fibrils [11]. The extraction process includes chemical hydrolysis and enzymatic hydrolysis. Hydrolyzed collagen is a group of peptides that can be extracted using different processes depending on the origin of the raw material [4,22,23]. Hydrolyzed collagen has higher solubility compared to native collagen and the extraction of hydrolyzed collagen is simple.

The most common animal collagen sources are human collagen, bovine, porcine, and marine organisms such as fish [24]. Despite the risk of bovine spongiform encephalopathy (BSE), bovine collagen is widely used in collagen-based products. The immune response to bovine collagen has been studied. A clinical study of 705 patients treated with a bovine collagen implant and a small percentage of patients had both the cellular and humoral types of the immune response [25]. Keefe, et al. showed that side effects to bovine collagen implants occurred in a small percentage of treated patients and these adverse reactions were resolved as the implant was resorbed by the patients [26]. Angiotensin-I converting enzyme (ACE) plays a key role in elevating blood pressure, and therefore, effective ACE inhibition can reduce blood pressure [27]. Bovine collagen is a promising precursor of ACE-inhibitory peptides in silico and in vitro protein digestions [28,29]. The porcine collagen is widely used for extracting collagen as the bovine collagen. Porcine collagen does not cause much allergic response because it is almost similar to human collagen. Collagen peptides are promising for osteoporosis treatment and prevention. Porcine collagen peptides could be used to treat and prevent osteoporosis [30]. Porcine collagen membrane was tested in vitro and in vivo studies for guided bone regeneration [31]. The result indicated that the use of the porcine collagen membranes did not cause foreign body reactions and the porcine collagen membranes were biocompatible.

In addition, collagen extracted from chicken feet merits special attention because they have essential health-beneficial nutrients. As with the other collagens, the collagen from chicken feet showed odors, water absorption, and texture characteristics as commercial material [32]. Chicken gelatines are a suitable alternative to those made from mammals or fish and have many pharmaceutical and biomedical applications [33]. Collagen type I can be also extracted from the ovine tendon to fabricate scaffold for tissue engineering applications [34]. The enzymatic hydrolysis of collagen from sheepskins at different times of hydrolysis was investigated [24]. The in vivo application, isolation, and characterization of acid-soluble goat tendon collagen in a murine wound was investigated. The results showed that the goat tendon collagen had comparable physicochemical properties with calfskin collagen and was significantly better cytocompatible material than collagen of bovine origin [35].

Collagen has porcine and bovine origins that cow and pig skins and bones are the main sources of collagen. However, due to religious constraints concerning the avoidance of porcine and bovine products or other reasons such as the outbreak of BSE, the marine collagen source is being highly considered by the industry as an important alternative [11,36,37]. Marine collagens have the advantages of a high yield and no disease transmission risk that can be obtained from invertebrate marine animals or fishes. Mammalian collagens have higher thermal stability than fish collagens due to the lower imino acid contents of fish collagens compared with mammalian collagens [38]. The thermal stability is related to body temperature and living environment and the low thermal stability of marine collagens restricts its applications [39]. The mechanical strength of marine collagen is poorer than collagen extracted from bovine because it is less crosslinked. After the crosslinking treatment, marine collagen can be used as a biomaterial in tissue engineering [40]. Despite some limitations, the marine collagen is an appealing option for product developers because the source is cheap and no risk of BSE. In the latest 20 years, more than 175 chemical entities and 28 marine natural products were discovered. Marine organisms, as well as their wastes, are good sources of collagen for many applications [41,42,43,44,45].

The functions of each type of collagen are briefly listed in Table 1. Collagen types I, II, and III are three collage types used for supplements. Collagen type I is mainly found in marine collagen. Collagen type II is original from chicken collagen and bovine collagen. A mixture of collagen type I and type III can be obtained from porcine collagen and bovine collagen. So far, there are many collagen products on the market. The most popular products may be collagen supplements as healthy food and cosmetic goods. The big market of collagen products indicates the significance of the effects of these products.

Many studies discussed the low immunogenicity of collagen. The low immunogenicity of porcine and bovine collagen type I were verified in vitro data and their suitability was favored for tissue-engineered scaffolds [46]. A study also investigated the biocompatibility and immunogenicity of two collagen micro/nanofiber materials, self-assembled collagen nanofiber and electrospun collagen nanofiber, that were prepared by tilapia skin collagen [47]. The evaluation demonstrated good biocompatibility and low immunogenicity for both collagen nanofiber materials according to tests of cytotoxicity, hemolysis, skin sensitization, acute systemic toxicity, mouse immunization, and lymphocyte proliferation.

## 3. Effects of Treatment

### 3.1. Skin Regeneration

The main component of the skin is collagen, and there are 85–90% collagen type I and 10–15% collagen type III. Collagen fibers that are damaged over time are strongly related to skin aging. Skin aging is caused by decreased collagen density and dermal thickness, as well as decreased synthesis and replacement of important structural proteins [48]. Collagen supplements originating from various sources such as marine, bovine, and porcine can improve skin integrity and modulate skin aging. They are effective in wrinkle reduction, skin rejuvenation, and skin aging reversal. Due to its high biocompatibility with the human body, collagen type I is the most used in cosmetic production. Oral collagen supplementation has become very popular in recent years. The effects of oral collagen supplementation on skin hydration and the dermal collagen network were studied [49,50]. A randomized, placebo-controlled, blind study of 72 healthy women over 35 years was performed to test the effect of a drinkable nutraceutical collagen product. Half of them (*n* = 36) received this collagen supplement that is a blend of 2.5 g of collagen peptide, biotin, vitamin C, acerola extract, zinc, and natural vitamin E complex for twelve weeks. Another half of the subjects received a placebo. The test product markedly improved skin hydration, roughness, elasticity, and density of the experiment group compared to the control group [51].

Kim et al. designed a randomized, double-blind, placebo-controlled trial to evaluate the efficacy of low-molecular-weight collagen peptide (LMWCP) with a tripeptide (Gly-X-Y) content on human skin hydration, wrinkling, and elasticity [52]. Compared with the placebo group, the LMWCP group had higher skin-hydration values after 6 weeks and 12 weeks, and two parameters out of three of the skin elasticity in the LMWCP group were significantly higher after 12 weeks. In addition, during the study period, none of the subjects presented adverse symptoms. These results suggested that LMWCP could be used as a healthy food ingredient to improve human skin conditions.

Marine collagen is becoming popular for maintaining skin health. Evans et al. performed a randomized, placebo-controlled, blind study to evaluate the efficacy of hydrolyzed marine collagen on skin health in women between 45 and 60 years of age. The results supported that the use of fish-derived hydrolyzed collagen could improve skin health in an aging population [53].

Asserin et al. investigated the efficacy of oral collagen peptide supplementation on skin hydration in a clinical study [49]. After several weeks of intake, the oral collagen peptide supplementation remarkably increased skin hydration, the collagen density in the dermis remarkably increased and the fragmentation of the dermal collagen network remarkably decreased. These effects persevered after 12 weeks. The results suggested that oral collagen peptide supplementation significantly improved skin aging.

Tanaka et al. examined the effect of daily intake of collagen peptides on the skin damage caused by repeated UV-B irradiation [54]. Intake of collagen peptide suppressed UV-B-induced skin hyperplasia of the epidermis, hydration decreases, and soluble type I collagen decreases. These results suggested that collagen peptides could suppress UV-B-induced skin damage.

### 3.2. Bone Defects

Tissue engineering is a biomedical engineering discipline that can replace or repair defective tissues with natural or synthetic tissue mimics. It aims to develop biological substitutes to replace, restore, or regenerate damaged tissues. Scaffolds, cells and growth-stimulating signals are the important components of engineered tissues. Scaffolds that are usually made of polymeric biomaterials can be used as an artificial structure to support three-dimensional (3D) tissue formation. They can provide structural support for subsequent tissue development and cell attachment [55].

Aging can lead to a reduction of all human capabilities. Loss of muscle or bone mass that causes sarcopenia, osteopenia, or osteoporosis with advancing age are major public health problems for the elderly population [56]. Bone loss has greatly affected elderly people. The bone-related medical treatments and costs are increasing in the aging population. Bone consists of a mineral phase (calcium phosphate) and an organic phase (collagen). The challenge in bone tissue engineering is to develop scaffolds having good biological and biomechanical properties [57]. Scaffolds can promote bone formation by differentiating towards the osteogenic lineage or releasing specific soluble molecules [58]. Scaffold architecture is very important for bone regeneration and acellular materials should allow proper host cell colonization for bone regeneration. In addition, mean pore size is another key component for proper cell colonization. Collagen-glycosaminoglycan scaffolds showed a substantial improvement in pore size [59].

A major challenge in clinical orthopedics is the regeneration of large segmental bone defects. The collagen scaffolds have been increasingly used as bone substitutes through tissue engineering approaches [60]. Preparation of collagen catecholamines and calcium composite structures was reported and the collagen composite scaffolds displayed outstanding mechanical properties [61]. These multifunctional scaffolds could be utilized to regenerate and repair bone defects. Nevertheless, the low mechanical strength of collagen limited its wider application in the field of bone regeneration. By combining different biological materials, the porosity, structural stability, osteoinduction and osteogenic properties of the collagen matrix can be greatly improved [40]. For example, a collagen scaffold loaded with human umbilical cord-derived mesenchymal stem cells was fabricated and applied for endometrium regeneration [62].

An alveolar cleft is a bone developmental defect and cancellous autologous bone harvested from the iliac crest is usually used for alveolar cleft repair [63]. Since this procedure has several drawbacks, bone substitutes were being used for alveolar cleft repair [64]. However, because the use of bone substitutes had not shown an advantage compared with autologous bone grafts, searching for novel bone substitutes is still necessary [65]. Collagen type I-based bone substitutes are good alternatives because collagen type I is a major component of bone. However, conventional collagen scaffolds obtained from xenogeneic tissues might increase the risk of unknown pathogens [66,67,68]. A recombinant collagen peptide (RCP) using human collagen type I was developed [69]. Compared to low or high cross-linked RCP particles, the medium cross-linked RCP particles had a better environment prone to generate bone tissue [70]. In addition, the effect of an octacalcium phosphate/weakly denatured collagen scaffold was investigated in a canine model [71]. The result showed it had better effect than the weakly denatured collagen scaffold.

### 3.3. Sarcopenia

The term, sarcopenia, was first introduced to refer to muscle wasting of older people [72]. And then in 2010, the definition of sarcopenia was altered to be low muscle mass together with low muscle function [73]. A patient can be diagnosed as sarcopenia when he/she has a slow walking speed and has muscle mass at least two standard deviations below the average [74]. There are age-related sarcopenia (primary sarcopenia) and disease-related sarcopenia (secondary sarcopenia). The causes of age-related sarcopenia included systemic inflammation, loss of motor units innervating muscle, a decline in anabolic hormones, and oxidative stress [73]. Early diagnosis and lifestyle intervention are the keys to improving the prognosis of patients with sarcopenia. The lifestyle interventions include exercise and nutritional supplements. In nutritional supplements, collagen supplements are effective to improve the symptoms of sarcopenia.

The effect of post-exercise protein supplementation with collagen peptides compared with placebo on the muscle mass and muscle function following resistance training of elderly sarcopenia patients was studied [75]. Additional dietary protein can increase the muscle protein synthesis rate after exercise and reduce the decomposition of muscle protein after resistance exercise. A total of 53 men with a mean age of 72.2 were recruited in the study. The results showed that 60 min of resistance exercise, performed three times per week, could significantly increase muscle mass, muscular strength, and motor control in sarcopenia patients. In addition, the study also showed that the combination of resistance exercise and collagen peptide supplementation resulted in a significant improvement in muscular strength as well as a significant increase in muscle mass and decrease in fat mass compared to placebo.

A study examined the effect of blood flow restriction (BFR) training with an additional post-exercise collagen hydrolysate supplementation on muscle mass and function in older men at risk of sarcopenia [76]. The study recruited 39 healthy men aged 50 years or older, and they were randomly assigned to one of three groups: low-load BFR training with protein (collagen hydrolysate), low-load BFR training with placebo, or a control group without training, but with protein supplementation. The results demonstrated that the addition of collagen hydrolysate showed a positive trend of increasing muscle mass and strength, but further research is needed to verify these effects with a larger sample size.

### 3.4. Wound Healing

Different types of wounds, such as ulcers and burns, may seriously influence the life quality of patients, especially chronic wounds that include pressure ulcers, diabetic foot ulcers, venous leg ulcers, and so on. Pressure ulcers are injuries to the skin and underlying tissue caused by prolonged pressure on the skin. Diabetic foot ulcers are a common complication of diabetes mellitus patients who are not well controlled. Venous leg ulcers are a pain in the legs usually due to weak blood circulation in the limbs. Chronic wounds may lead to significant morbidity and poor quality of life. The chronic wounds may not be treated by the normal therapy, usually because of an overactive and prolonged inflammatory response, altered protease levels, and deficient ECM [77]. As a result, advanced wound therapies are under development. Collagen is one of the biomaterials that are very useful for developing advanced therapies. The wet strength of collagen sponges allowed soft tissue to be sutured and provided a template for the new tissue growth. Collagen-based implants have been used as carriers for the delivery of drugs for skin replacement and burn treatment [78].

Collagen has been used for wound care as a wound dressing material for a long time. Ancient Egyptians and Greeks used honey, silk, and lint as materials in wound management. It was very little changed until the late twentieth century that polymer dressings were discovered to accelerate the rates of wound repair and re-epithelialization [79]. Traditional dry dressing treatments, such as absorbent cotton and absorbent gauze, might not have a good therapeutic effect. On the contrary, a moist healing environment was conducive to the growth of granulation and the division of skin cells, thereby accelerating the healing of the wound. Collagen hydrogel was demonstrated as a potential wet wound dressing material that could significantly accelerate the generation of new skin appendages [78]. Collagen dressings were usually formulated with bovine, avian, or porcine collagen and were easy to apply and remove [80]. In addition, collagen dressings could be derived from the marine source. Nile tilapia is one of the most popularly cultured fish in China and collagen hydrogels from tilapia skins could be used as wound dressings for the treatment of deep second-degree burns [81].

Collagen oral administration could be also an efficient treatment for wound healing. A study investigated the effect of oral administration of the collagen peptides derived from jellyfish in wound healing [82]. It was shown that collagen peptides from Jellyfish-Rhopilema esculentum might be beneficial to wound clinics in the future due to their good characteristic in accelerating the wound healing process. The wound healing potential of oral administering collagen peptides from chum salmon skin in wound rat models was investigated [83]. Oral administration of marine collagen peptides derived from chum salmon improves wound healing in rats [84]. The result showed the efficacy of oral administering collagen peptides treatment on wound healing in animals. The oral application of specific bioactive collagen peptides has also demonstrated positive effects on wound healing. A study showed that would patients treated with bioactive collagen peptides had a better outcome compared with the placebo groups [85]. A collagen-derived peptide, prolyl-hydroxyproline (Pro-Hyp), was a growth-initiating factor for specific fibroblasts involved in the wound healing process [86].

### 3.5. Dental Therapy

Periodontitis is a highly prevalent disease among adults that is usually triggered by a bacterial infection and it affects the tissues surrounding the dentition. If left untreated, it can lead to loss of teeth. The use of different biomaterials in periodontal regeneration has been studied for years. Several treatment options for periodontal disease include open flap debridement (OFD), biologically active regenerative materials, bone-replacement graft materials, scaling and root planning, and guided tissue regeneration with barrier membranes [87,88,89,90,91,92]. The combined use of barrier membranes and biomaterials was shown to be more effective than using OFD alone. Collagen from either human or animal tissue was widely used as natural resorbable barrier membranes and the resorption of collagen membranes depended on the source of the material (bovine, porcine, human) [93].

Tizzoni and Tizzoni illustrated a case of how a tooth was preserved through a periodontal regeneration surgery [94]. The use of two equine collagen membranes associated with an equine bone graft carried out periodontal regeneration according to guided tissue regeneration principles. This case showed that the use of collagen membranes could improve bone regeneration at the defect site. Besides, a scaffold was generated by electrospinning a basic poly-lactic-co-glycolic acid and polycaprolactone matrix, followed by silver nanoparticles impregnation, polydopamine coating, and then coating by collagen I [95]. In a mouse periodontal disease model study, this scaffold was effective for periodontitis treatment by enhancing alveolar bone regeneration.

Gingival recession (GR) is the exposure of root surfaces that is caused by apical migration of the gingival tissue margins and is frequently detected in adults [96]. The reference therapy for GR is the connective tissue graft (CTG) plus coronally advanced flap (CAF). Collagen bioscaffold was used for the treatment of GR [97]. For GR, cell adhesion in the scaffold influences the integration, migration, and survival of the cells among the scaffold. Collagen was found to be a superior bioscaffold for the treatment in GR. In a prospective randomized, controlled 18 adult patients clinical trial, a porcine collagen matrix plus CAF treatment showed no statistically significant differences compared with the CTG plus CAF treatment evaluated at 12 months [98]. As a result, the collagen matrix could be a possible alternative to CTG. Moreover, due to aging or disease, dentinal collagen undergoes glucose-related crosslinking. However, it remains unknown how collagen crosslinking affects the early adhesion of related oral streptococci. Schuh et al. studied the effect of glycated collagen on oral streptococcal nanoadhesion to help develop a new preventive and therapeutic treatment against dental caries [99].

In addition, tooth extraction may cause postoperative complications, and therefore, the management of the post-extraction healing is important. The device recombinant human bone morphogenic proteins, along with an absorbable collagen sponge as a carrier, was successfully used for ridge preservation procedures after tooth extraction [100]. The extraction of the third molar is the most frequent oral surgery that sometimes is associated with severe or minor complications. A retrospective study evaluated the postoperative complication rates for the use of absorbable collagen type I sponge in third molar extraction [101]. A total of 3869 third molars in 2697 patients were extracted and the extraction sockets were packed with collagen type I sponges. The study showed a relatively low incidence of complications in the use of collagen type I sponge [102]. The result showed that it could help relieve pain, reduce the frequency of mouth-opening limitation, and increase the mineralization ratio at the extraction socket site.

Moreover, in a prospective observational (non-controlled) clinical study, 15 patients went through a keratinized tissue reconstruction around dental implants with a porcine collagen matrix [103]. After 6 months and 1, 4 and 5 years of evaluation, a porcine collagen matrix was shown to have good efficacy in keratinized tissue augmentation. As a result, the porcine collagen matrix integration can be a good scaffold to regenerate keratinized mucosa ensured perfect healing.

### 3.6. Gastroesophageal Reflux

Gastroesophageal reflux (GERD) is a digestive disorder that occurs when acidic stomach fluids back up from the stomach into the esophagus. GERD is a risk factor of esophageal cancer [104] and is associated with major depression [105]. About half of adults experience reflux symptoms sometimes [106]. To identify GERD-associated genes, four separate patient cohorts were examined using the genome-wide linkage, gene association, and protein level analyses [107]. Collagen type III alpha 1 gene was identified to be associated with GERD. In addition, the hiatal hernia plays a role in the development of both acid reflux and GERD. The prevalence of GERD with hiatal hernia can reach 94%. Diemen et al. showed that the composition of phrenoesophageal ligament (POL) for patients with GERD and hiatal hernia included less total, type-I and type-III collagens than that of the phrenoesophageal ligament of cadavers without hiatal hernia [108].

Pharmaceutical and surgical treatments have been developed for GERD. However, pharmaceutical medications can only reduce GERD symptoms and may cause serious side effects, while surgery is invasive. As a result, the development of various endo-luminal outpatient therapies for GERD is a more attractive option. Traceback to 1988, the collagen treatment for human GERD was investigated [109]. Ten patients with severe refractory reflux symptoms were treated with endoscopic technique. Cross-linked bovine dermal collagen was injected under the mucosa in the lower esophageal sphincter area. All patients developed objective evidence of decreased reflux. This endoscopic implant treatment resulted in statistically significant improvement.

A composite material composed of round and smooth polymethyl methacrylate (PMMA) microspheres suspended in a collagen “carrier”, called Collagen/PMMA implant (G125), had shown a promising endoscopic ‘bulking agent’ for treating GERD due to its proven tissue biocompatibility and persistence [110]. G125 can achieve permanent and submucosal lower esophageal sphincter soft tissue augmentation and the bovine collagen carrier material can prevent the microspheres from both migrating and agglomerating during the critical one-month tissue remodeling phase after injection. Therefore collagen scaffold played an important role in the implant injection in the treatment of GERD [111].

### 3.7. Osteoarthritis

OA is one of the most common joint diseases caused by the breakdown of joint cartilage and underlying bone and is a significant cause of disability [112]. OA is understood to be a complex interaction of systemic and local factors [113]. Many pharmaceutical and nutraceutical agents have been developed to delay the progression of OA cartilage structural changes. Collagen type II is the main component of the cartilage tissue and is the potential to be used as a treatment of OA [114,115]. A randomized controlled trial with 39 patients diagnosed with knee OA was included and was randomly distributed into two groups: one treated with acetaminophen (*n* = 19) and the other treated with acetaminophen plus type II collagen (*n* = 20) for 3 months [116]. The result showed that the type II collagen treatment combined with acetaminophen was superior to the only acetaminophen treatment.

Undenatured type II collagen (UC-II) is a nutritional supplement extracted from chicken breast cartilage. A multicenter double-blind, randomized, placebo-controlled study was performed to compare UC-II with placebo and with glucosamine hydrochloride plus chondroitin sulfate (GC) [117]. On day 180, the UC-II group demonstrated a significant reduction in overall Western Ontario McMaster Universities Osteoarthritis Index score compared with placebo and GC.

Collagen peptides (CP) are used as a component of nutraceuticals. Isaka et al. studied the effect of CP on the articular cartilage of OA using a rat experimental OA model by evaluating the serum levels of biomarkers, histopathological changes, type II collagen, immunohistochemical staining of matrix metalloproteinase [118]. The observations suggested that CP may exert chondroprotective action on OA by inhibiting the expression of matrix metalloproteinase-13 and type II collagen degeneration. It was reported that oral consumption of type 1 hydrolyzed collagen (hCol1) can relieve the pain in human OA. Dar et al. studied the effect of orally administered hCol1 in a model of posttraumatic OA [119]. Significant chondrocyte and cartilage and effects were observed in the degenerative knee of mice supplemented with hCol1. The results suggested that hCol1 was anti-inflammatory and chondroprotective in posttraumatic OA.

In OA cartilage, hypertrophic differentiation can be observed in degenerative chondrocytes [120,121]. Collagen type II is an important signaling molecule that can regulate chondrocyte proliferation, differentiation, and metabolism [122]. The collagen type II decrease can cause chondrocyte hypertrophy in OA cartilage. In a mouse model, loss of collagen type II was found to promote chondrocyte hypertrophy via the bone morphogenetic protein (BMP)-SMAD1 pathway [123]. This result revealed the inhibition mechanisms of chondrocyte hypertrophy by collagen type II and suggested that the degradation in collagen type II might initiate and promote OA progression. Crowley et al. evaluated the effectiveness and safety of UC-II compared with a combination of glucosamine and chondroitin (G + C) in the treatment of the knee OA [124]. At the end of 90 days of treatment, UC-II treatment reduced an index score by 20%, compared with 6% in the G + C treatment group. The daily activities of the UC-II group were significantly enhanced.

Collagen is a good treatment candidate for OA among the different therapeutic options due to its safety and clinical evidence. Two different approaches for collagen include collagen hydrolysates and native collagen and both types of collagen nutraceuticals are effective in reducing OA pain, in animal models and human clinical trials. Native collagen, which may be poorly absorbed, could work through a mechanism of oral induction, and hydrolyzed collagen can reach the target site where collagen synthesis is needed. As a result, collagen represents a good therapeutic option to improve OA [15].

### 3.8. Rheumatoid Arthritis

RA is a chronic debilitating autoimmune and inflammatory disorder that mainly affects joints. RA causes cartilage and bone erosion by invading fibrovascular tissue [125]. Preliminary studies suggested that oral administration of cartilage-derived collagen type II was clinically beneficial and safe in patients with RA. Two hundred seventy-four patients with active RA were randomized to receive placebo or different dosages of oral cartilage-derived collagen type II for 24 weeks [126]. At the lowest tested dose, positive effects were observed in the collagen treatment group, and the therapeutic agent had no side effects.

A 24-week randomized methotrexate-controlled study was conducted to evaluate the safety and efficacy of chicken collagen type II (CCII) in the treatment of RA [127]. Chicken collagen type II is a protein extracted from chicken breast cartilage. It has the potential to treat autoimmune diseases by inducing oral tolerance. Chicken type II collagen is a protein extracted from chicken breast cartilage. It has the potential to treat autoimmune diseases by inducing oral tolerance. Five hundred three RA patients were randomized into two groups. The result showed that CCII was effective in the treatment of RA and was safe for consumption. In addition, the development of therapeutic DNA vaccines might provide new promising strategies for the treatment of RA [128]. A new therapeutic DNA vaccine encoding CCII (pcDNACCOL2A1) was developed and a single injection of the pcDNA-CCOL2A1 vaccine alone could induce strong immune tolerance against experimental RA [129]. As a result, this vaccine might have therapeutic applications in the treatment of RA and appears to be as effective as the current “gold standard” treatment of methotrexate. The immunogenicity and safety of the pcDNA-CCOL2A1 vaccine in Wistar rats were investigated [14]. The results showed that at the maximum dosage of 3 mg/kg, the pcDNA-CCOL2A1 vaccine was well-tolerated and safe.

**Figure 2:**
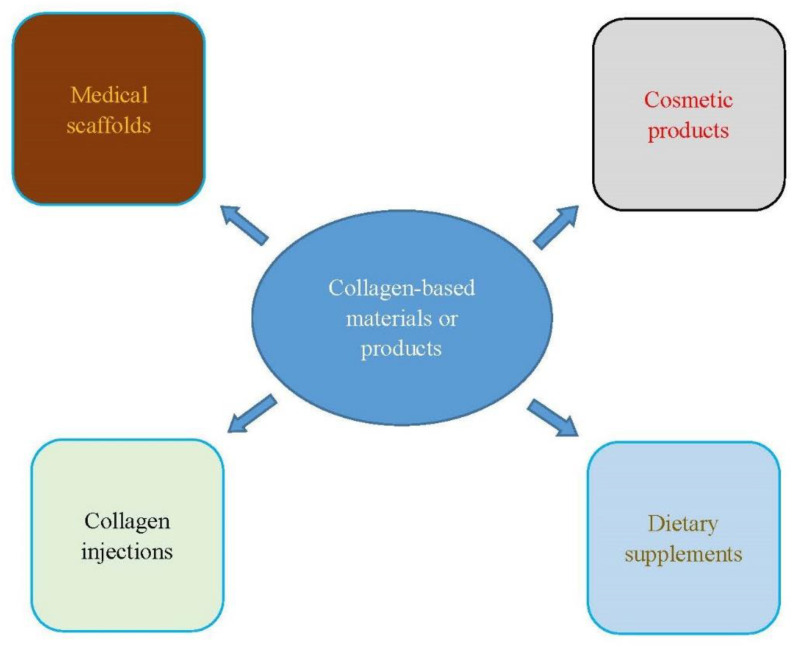
The functions of collagen-based biomaterials or products.

The collage treatments reviewed in this section are summarized in Table 2. Collagen-based biomaterials or products can be used to treat more diseases or symptoms than those discussed in this paper. Figure 2 provides the functions of collagen-based biomaterials or products.

## 4. Discussion

### 4.1. Treatment of Comorbid Diseases and Preventing Complications

The collaged-based treatments for skin regeneration, bone defects, periodontal disease, GERD, OA and RA have been discussed in Section 3, respectively. The association between some of these diseases has been investigated in the literature. RA was associated with skin disease. Dermatologic involvement usually occurred in patients with more severe RA [130]. Cutaneous manifestations might have a notable negative impact on the emotional, physical, and psychosocial health of RA patients [131]. Knowing the cutaneous expressions of RA may lead to early diagnosis, timely treatment, and lower morbidity for the patients [132]. Mesenchymal stem cells (MSCs) have been studied as a treatment for OA. Exosomes isolated from various stem cells may help tissue regeneration in the skin and inhibit the development of OA [133]. The prevalence of comorbidities among female patients with generalized OA was investigated [134]. Female patients with hand and knee OA were invited to participate in the study including two hundred generalized OA and two hundred control participants. GERD was observed to be one of the comorbidities of generalized OA. A 55-year-old female patient with endosseous dental implants had GERD and OA [135]. A possible connection between GERD and RA was discussed [136]. Miura et al. conducted a study to explore the relationship between RA and GERD. Two hundred and eleven patients in Japan were studied. The prevalence of GERD in RA patients was significantly higher than that in the Japanese population [137]. The prevalence of GERD symptoms in RA patients was high, and it is closely related to decreased functional status [138]. A total of 1147 patients with RA and 5735 controls were included in a study to investigate the association between RA and OA [139]. All participants were retrospectively traced, up to 14 years. Multivariate logistic regression analyses showed that patients with symptomatic OA had a significantly higher risk of RA. The high prevalence of secondary OA in patients with RA was determined in the trial [140].

In addition, collagens could modulate bone fracture callus formation. A mice model study demonstrated that diminished collagen type III leads to decreased bone formation and alterations in remodeling during fracture healing [141]. Thereby, collagen type III may play an important role in modulating the repair process in fracture. There are often fractures and contusions in the injuries caused by a car accident, falls or sports. Collagen oral administration is an efficient treatment for wound healing. As a result, collagen could be a very important nutritional supplement for those who suffered from fractures and contusions in the injuries caused by accidents.

Collagens are involved in the pathogenesis of many diseases. The associations between these disorders may reveal that the comorbidity of these diseases might be closely related to collagen deficiency. It indicates that collagen treatment might be a good choice when a patient has more than one of these diseases at the same time. It suggests that collagen therapy can be used in treating comorbid diseases and preventing complications.

### 4.2. Collagen and COVID-19

**Figure 3:**
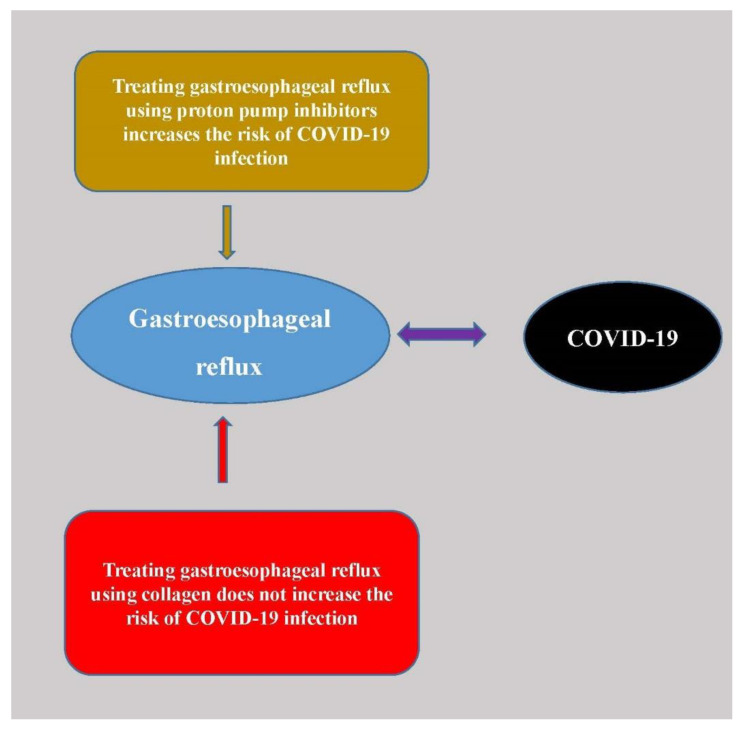
Using collagen to treat gastroesophageal reflux does not increase the risk of COVID-19 infection as the proton pump inhibitors drug.

In addition to the above mentioned diseases, collagen may be related to the treatment of COVID-19. COVID-19 was caused by severe acute respiratory syndrome coronavirus 2 (SARS-CoV-2) and it has infected more than 173 million people all over the world, that has had a huge impact on people’s lifestyle, economy, and livelihood. COVID-19 was first identified in Wuhan, China in December 2019 and then spreads to Europe, the USA and other countries [142]. The severity of COVID-19 symptoms can range from mild to severe. Many patients died from COVID-19 due to serious complications or COVID-19 related lung diseases. COVID-19 patients with some chronic diseases such as diabetes, cardiovascular diseases, hypertension, and malignancies may develop a critical situation [143]. GERD was also reported to be correlated with COVID-19. Proton pump inhibitors (PPIs) are a class of GERD drugs that can be used to reduce stomach acid and relieve GERD symptoms. Evidence showed that the use of PPIs was correlated to COVID-19 infection [144,145]. Individuals taking PPIs twice daily had a higher risk for COVID-19 infection compared with those using lower-dose PPIs up to once daily [145]. The prevalence of laryngopharyngeal reflux disease (LPRD) may be higher than the general population, which indicated that COVID-19 impairs the upper esophageal sphincter and aggravate reflux. A retrospective study of 95 hospitalized patients with COVID-19 showed that the patients with laryngopharyngeal reflux disease (LPRD) were correlated with poorer clinical outcomes [146]. They also concluded that COVID-19 could impair the upper esophageal sphincter and aggravate reflux. According to these studies, it may conclude that GERD could cause poorer outcomes of COVID-19 and the commonly used GERD drug PPI might not be suitable to be used in COVID-19 patients. Although there are some other drugs for GERD, collagen has been shown to have a good effect in treating GERD. In addition, collagen supplements appear to be safe and have fewer side effects than other GERD drugs (Figure 3). As a result, collagen supplements could be a very good choice to be used in COVID-19 patients with GERD. In addition, Zhu et al. studied how antibodies interacted with the structure of collagen and the extracellular matrix [147]. They discovered a histocompatibility complex recognition region within collagen that may help protect the body from invading viruses. The discovery may be relevant to the treatment of COVID-19, wherein the body attacks its own collagenous tissues. So far, research on the association between collagen therapy and COVID-19 has been very limited. Nevertheless, taking collagen supplements or collagen-rich foods has been proven to have many benefits. As a result, the collagen supplement may be used as a nutritional product for COVID-19 patients to reduce the risk of other complications.

### 4.3. The Disadvantages of Collagen Treatment

From the above discussions, compared with other drugs, collagen treatment has many advantages. However, it does not mean that the collagen treatment is absolutely safe. Some people may have an allergic reaction to collagen treatment. For example, some people may have a shellfish allergy and could experience anaphylaxis if they take marine collagen supplements. The other source of collagen may have an allergy problem.

The bovine type I collagen is the main content in the gelatin contained in the measles, mumps, and rubella vaccines. Fish meat and skin also contain type I collagen. Most children with anaphylaxis showed sensitivity to these vaccines that might be due to the bovine gelatin that was included in the vaccines [148]. Two cases of allergic reaction to bovine collagen as a therapeutic device or treatment, including a 39-year-old woman and a 62-year-old woman, were reported [149].

**Figure 4:**
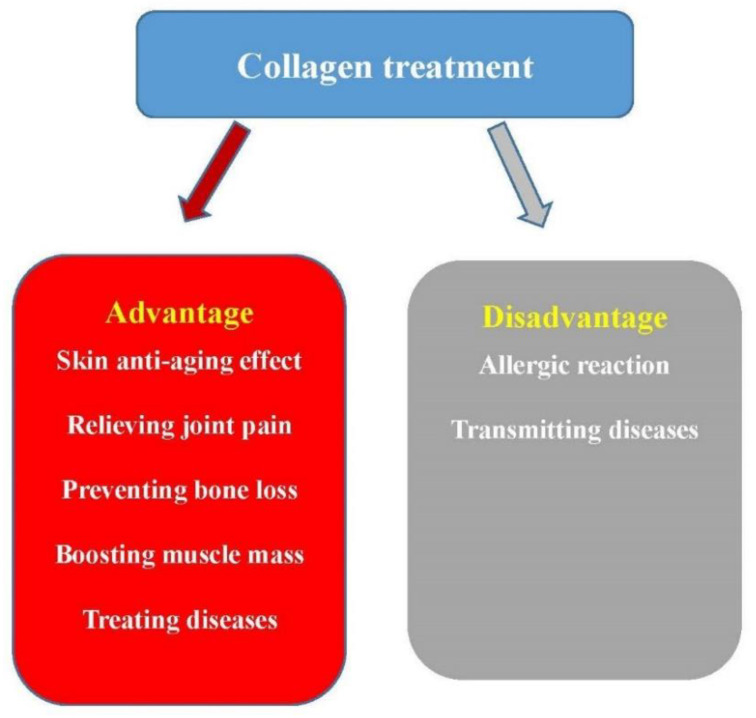
Some advantages and disadvantages of the collagen treatment.

In addition, animal collagen sources have the risk of transmitting diseases. Porcine and bovine origin collagen carries a risk of transmitting illnesses such as bovine BSE. BSE risk induced by ruminant collagen and gelatine produced from raw material for human consumption has been investigated [150]. Bovine-derived graft materials were also widely used in dental surgery and might carry a risk of prion transmission to patients [151]. If this disadvantage of transmitting diseases for porcine and bovine origin collagen is considered, as we discussed in Section 2, marine collagens could be good substitutes. Figure 4 lists some advantages and disadvantages of collagen treatment.

## 5. Conclusions

Collagen is the most abundant protein in the human body that has many multi-functions. The loss or defect of collagen can cause skin aging and other diseases. The collage treatments have demonstrated effective improvements in skin hydration, skin elasticity, medical scaffold treatment, GERD, OA and RA in many clinical studies. In addition, the collagen treatment for GERD in COVID-19 patients is also discussed in this study. Collagen therapy can reach good improvement and does not cause any serious adverse reactions. Collagen-based materials and products are the potential to be used in more applications, and they are the one of most important supplements for aging people.

## Figures and Tables

**Table 1:** The function of the 5 most common types of collagen.

Collagen	Function or Application	Tissue or Organ	Molecular Composition *
Type I	the organic part of the bone, membranes for guided tissue regeneration	Skin, bone, teeth, tendon, ligament, vascular ligature	${\left[\alpha 1(I)\right]}_{2}\alpha 2(I)$
Type II	the main constituent of cartilage, cartilage repair, and arthritis treatment	cartilage	${\left[\alpha 1(\mathrm{II})\right]}_{3}$
Type III	the main constituent of reticular fibers, hemostats, and tissue sealants	muscle, blood vessels	${\left[\alpha 1(\mathrm{III})\right]}_{3}$
Type IV	the major component of the basement membrane, attachment enhancer of cell culture, and diabetic nephropathy indicator	basal lamina, the epithelium-secreted layer of the basement membrane	${[\alpha 1(\mathrm{IV})]}_{2}\alpha 2(\mathrm{IV})$ ${[\alpha 3(\mathrm{IV})]}_{2}\alpha 4(\mathrm{IV})$ ${[\alpha 5(\mathrm{IV})]}_{2}\alpha 6(\mathrm{IV})$
Type V	feedstock for biomaterials in corneal treatments	Hair, cell surfaces, and placenta.	α1(V),α2(V),α3(V)

* α1(I),α2(I),α1(II),α1(III),α1(IV),α2(IV),α3(IV),α4(IV),α5(IV),α6(IV),α1(V),α2(V), and α3(V) are proteins encoded by COL1A1, COL1A2, COL2A1, COL3A1, COL4A1, COL4A2, COL4A3, COL4A4, COL4A5, COL4A6, COL5A1, COL5A2 and COL5A3 genes, respectively.

**Table 2:** The references of the collage treatments.

Disease	Collagen Treatment References
Skin aging	[48,49,50,51,52,53,54]
Bone defects	[41,58,59,60,62,64,65,66,67,68,70,71]
Sarcopenia	[75,76]
Wound healing	[78,79,80,81,82,83,84,85,86]
Periodontal disease	[86,93,94,95,96,97,98,99,100,102]
Gastroesophageal reflux	[107,108,109]
Osteoarthritis	[15,112,113,114,115,116,117,120,121,122,124]
Rheumatoid arthritis	[14,125,126,127,128,129]

## Data Availability

Not applicable.
